# Effect of Donated Premature Milk in the Prevention of Bronchopulmonary Dysplasia

**DOI:** 10.3390/nu16060859

**Published:** 2024-03-15

**Authors:** Amaia Merino-Hernández, Andrea Palacios-Bermejo, Cristina Ramos-Navarro, Silvia Caballero-Martín, Noelia González-Pacheco, Elena Rodríguez-Corrales, María Carmen Sánchez-Gómez de Orgaz, Manuel Sánchez-Luna

**Affiliations:** Neonatology Division, Hospital General Universitario Gregorio Marañón, 28009 Madrid, Spain; apb8257@gmail.com (A.P.-B.); cristinaramosnavarro@yahoo.es (C.R.-N.); sylviamarina@hotmail.com (S.C.-M.); noelia.gonzalez.pacheco@gmail.com (N.G.-P.); elenarodriguezcorrales@gmail.com (E.R.-C.); msgomezdeorgaz@salud.madrid.org (M.C.S.-G.d.O.); msluna@salud.madrid.org (M.S.-L.)

**Keywords:** bronchopulmonary dysplasia, mother’s own milk, pasteurized donor milk

## Abstract

Introduction: Breastfeeding is one of the strategies that has been shown to be effective in preventing severe forms of bronchopulmonary dysplasia (BPD). When mother’s own milk (MOM) is not available, pasteurized donor milk (DM) is the best alternative. However, the evidence is inconclusive on the difference in the incidence of bronchopulmonary dysplasia (BPD) between patients fed MOM and those fed with DM. As standard DM is usually mature pooled milk donated by mothers who have delivered their babies at term, the potential benefits of preterm milk may be lost. Materials and Methods: An observational, retrospective, single-center study was conducted in the neonatology department of a high-complexity hospital. The study included newborns <32 weeks of gestational age born between January 2020 and December 2022. When supplemental milk was needed, non-pooled preterm pasteurized donor milk (PDM) matched for gestational age and moment of lactation was used in this study, classifying preterm infants in two groups: mainly MOM (>50% of the milk) or mainly PDM (>50% of the milk). Two groups were established: those who received >50% MOM and those who received >50% PDM. They were also classified according to the diagnosis of DBP: one group included no BPD or grade 1 BPD (noBPD/1), while the other included grade 2 or 3 BPD (BPD 2–3). The objectives of this study were, firstly, to evaluate the incidence of BPD 2–3 among patients who predominantly received PDM versus MOM. Secondly, to analyze differences in the type of human milk received and its nutritional components, as well as to study the growth in patients with or without BPD. Results: One hundred ninety-nine patients were included in the study. A comparison of noBPD/1 versus BPD 2–3 groups between those receiving mainly MOM versus PDM showed no significant differences (19% vs. 20%, *p* 0.95). PDM colostrum in BPD 2–3 compared to noBPD/1 was higher in protein content (2.24 g/100 mL (SD 0.37) vs. 2.02 g/100 mL (SD 0.29) *p* < 0.01), although the statistical significance decreased after adjustment for gestational age and birth weight z-score (OR 3.53 (0.86–14.51)). No differences were found in the macronutrients in the mature milk of patients feeding more than 50% PDM in both study groups. Growth of BPD 2–3 showed a greater decrease in the difference in z-scores for height at birth and at discharge compared to noBPD/1 (−1.64 vs. −0.43, *p* 0.03). Conclusions: The use of mainly MOM or PDM demonstrates a similar incidence of noBPD/1 or BPD 2–3. Non-pooled and matched by gestational age and time of lactation preterm donor milk can probably be an alternative when mother’s own milk is not available, with a similar protective effect in the prevention of severe BPD.

## 1. Introduction

Bronchopulmonary dysplasia (BPD) is the most common morbidity after preterm birth [1,2]. The incidence of this disease is higher the more premature the newborn is, with some studies reporting BPD of grade 2–3 in up to 55% of newborns aged less than 28 weeks [3].

As there is no effective treatment, prevention strategies are key for preterm infants at risk of BPD, and breastfeeding with mother’s own milk (MOM) is one of the strategies that has been shown to be effective in the prevention of severe forms of BPD [4,5,6]. This protective effect seems to be related, among others, to the effect of the components of breast milk in reducing oxidative stress, which plays a fundamental role in the inflammatory process and alveolar destruction related to dysplasia [6,7,8]. Therefore, there is strong support for the recommendation of MOM as the main source of nutrition, especially for very low birth weight (VLBW) infants [9,10].

However, the availability of MOM is often limited. In such cases, pasteurized donor milk (DM) seems to be the best alternative, with studies showing efficacy in reducing BPD compared to preterm formula [11,12].

The literature is less forceful on the comparison between MOM and DM feeding on the effect of BPD [11]. The pasteurization process can alter the components of human milk and reduce important protective elements such as lactoferrin, lysozymes, growth factors, and antioxidants [13,14]. In addition, DM is typically mature pooled milk from mothers given to their babies at term, exhibiting different components compared to the milk of mothers of preterm infants [15].

At present, there is not enough evidence that DM is as effective as MOM in reducing BPD. In this context, it is proposed that donor milk from mothers of preterm infants may have advantages compared to standard mature milk from a term mother used in standard milk banks. Therefore, in this study, donated pasteurized milk from mothers of non-pooled preterm infants classified by gestational age and breastfeeding time was used [16].

As current evidence regarding the potential benefits of standard DM in preventing BPD compared to MOM is primarily derived from studies utilizing pooled mature milk donated by mothers who have delivered at term, the objective of this study was to compare the incidence of BPD among premature infants receiving predominantly MOM versus predominantly pasteurized non-pooled preterm donor milk (PDM) matched by gestational age and time of lactation.

The secondary objective of this study was to analyze differences in the nutritional components of PDM in patients with or without BPD.

## 2. Materials and Methods

### 2.1. Study Design

An observational, descriptive, retrospective study was conducted in the Department of Neonatology of the Hospital General Universitario Gregorio Marañón in Madrid (HGUGM) between January 2020 and December 2022.

The HGUGM is a tertiary hospital (level IIIC) [17] with an average number of births per year of approximately 5000.

### 2.2. Participants

Inclusion criteria:

Newborns less than 32 weeks of gestational age born in our department or transferred to it in the first 24 h of life.

Exclusion Criteria:

Newborns who died during the first 14 days of life.

Newborns transferred to our unit with more than 24 h of age.

Newborns with insufficient information about the nutrition they have received.

Newborns with heart or lung malformations, metabolic diseases, or those syndromes that significantly affect growth or nutrition.

### 2.3. Data Collection

Epidemiological and clinical data were collected from neonates meeting the inclusion criteria. A database was created in which variables obtained from the hospital history of each patient were recorded, including sex, gestational age, weight, height, and head circumference at birth and their percentile and Z score according to intergrowth charts [18], intrauterine growth restriction (IUGR), small for gestational age (SGA), antenatal corticosteroids, chorioamnionitis, type of delivery, and type of nutrition (PDM or MOM). In the case of PDM, analyzed colostrum was recorded in all patients who received it, whereas analyzed mature milk was only recorded if the predominant type of nutrition was PDM (>50% of DM). The analysis of transitional milk was not included due to insufficient recorded data. Respiratory outcome variables included the need for surfactant administration, invasive mechanical ventilation (IMV), postnatal corticosteroids, and the duration and total days of oxygen therapy. Neonatal outcomes studied included the incidence of BPD, necrotizing enterocolitis (NEC), retinopathy of prematurity (ROP), grade III or IV intraventricular hemorrhage (IVH III/IV), sepsis, late-onset sepsis (LOS), and patent ductus arteriosus (PDA).

### 2.4. Grouping

Two groups were established: those who received >50% MOM and those who received >50% PDM. They were also classified according to the diagnosis of DBP: one group included no BPD or grade 1 BPD (noBPD/1), while the other included grade 2 or 3 BPD (BPD 2–3). This classification was used to compare the macronutrient content of the PDM they received, perinatal factors, and growth patterns between the two groups.

### 2.5. Fortification

Standard fortification with cow’s milk-derived fortifier was given in both groups.

### 2.6. Premature Donor Milk Bank

Pasteurized donor milk from mothers of premature infants matched by gestational age and days of life and adapted as much as possible to nutritional needs status was used. To this end, a classification is made according to the type of milk: colostrum (milk produced after delivery until 5 days of age), transition (up to 21 days of age), and mature (from 22 days of age); and also by gestational age: less than 28 weeks, between weeks 28 and 32, from week 32 to 37, and above week 37 of gestation [16].

### 2.7. Definition of Bronchopulmonary Dysplasia

BPD was defined as set by the NIH consensus in 2001 [19].

### 2.8. Statistical Analysis

A descriptive analysis was performed, using absolute frequencies and percentages for the analysis of qualitative variables and means with a standard deviation or medians with an interquartile range according to the symmetry of the distributions for the quantitative variables.

Proportions were compared using Pearson’s χ^2^ test and Fisher’s exact test, as appropriate, and quantitative variables were compared using Student’s *t*-test for normal distributions and Mann–Whitney’s U test for non-normal distributions. A multivariate analysis was performed using logistic regression. Statistical significance was established at *p* < 0.05. The statistical program IBM SPSS Inc., Armonk, NY, USA, version 25, was used.

### 2.9. Approval of the Ethics Committee

This study was approved by the Ethics and Drug Research Committee of the center where the work was carried out (code: HCPREVDBP 1.0).

## 3. Results

During the study period, 231 patients were born under 32 weeks, of whom 32 died, so a total of 199 patients were included.

**Figure 1:**
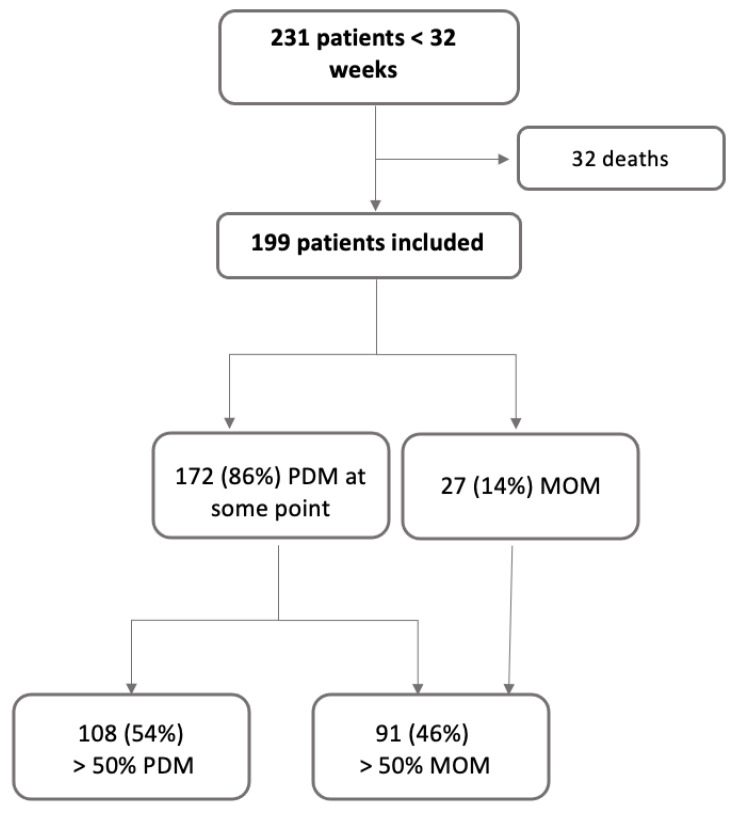
Flow chart of patients included in the study by predominant type of feeding. PDM: pasteurized preterm donor milk. MOM: mother’s own milk.

Of the total number of patients included in the study, 172 (86%) received pasteurized preterm donor milk at some point during their admission; 108 of these patients (54%) received more than 50% PDM, and 91 patients received more than 50% MOM (46%) (Figure 1).

**Figure 2:**
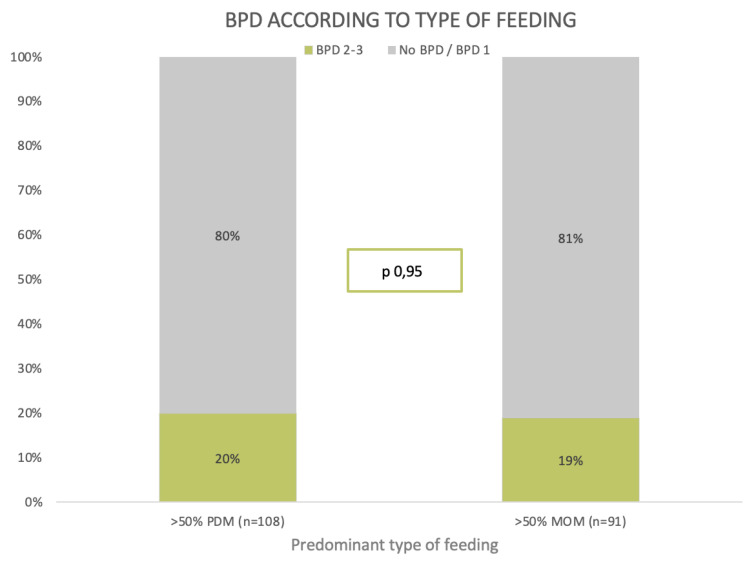
BPD by type of feeding. Comparison of proportions using chi-square. PDM: pasteurized preterm donor milk. MOM: mother´s own milk.

When comparing the diagnosis of BPD between patients who received predominantly MOM versus PDM, no differences were found between the two groups (19% vs. 20%, *p* 0.95) (Figure 2).

One hundred fifty-nine (79.9%) patients in the study were classified in the noBPD/1 group and 40 (20.1%) in the BPD 2–3 group. The characteristics of the sample are described in Table 1.

When analyzing the macronutrients of patients who had received colostrum from PDM, it was observed that the BPD 2–3 group received colostrum with a higher protein content than the noBPD/1 group (2.02 g/100 mL (SD 0.29) vs. 2.24 g/100 mL (SD 0.37), *p* < 0.01), maintaining this difference when adjusting for gestational age in a multivariate model (Table 2) but not when the birth weight Z score was included in the model (OR 3.53 (0.86–14.51)). When comparing the mature milk of patients who had been fed more than 50% with PDM, no differences in any of the macronutrients were found between the two groups, including the mean protein intake (Table 2) (PDM + fortification) (noBPD/1 4.48 (SD 1.01) g/kg/day vs. BPD 2–3, 4.92 (SD 1.10) g/kg/day, *p* 0.12), with no differences when adjusting for gestational age in a logistic regression model (OR 1.95 (1.00–3.80)).

When analyzing other nutritional variables related to BPD, it was observed that in patients with BPD 2–3, fortification was started later (16.62 days (SD 8.92) vs. 10.96 days (SD 6.91), *p* < 0.01), as well as exclusive enteral nutrition was achieved later (17.28 days (SD 14.14) vs. 10.34 days (SD 8.69), *p* < 0.01). This difference was not maintained when adjusting for gestational age in a multivariate model (OR 1.03 (0.98–1.10) and OR 1.01 (0.98–1.05), respectively). No differences were found between patients with BPD 2–3 and noBPD/1 patients in the mean total intake at 3 and 7 days (123.10 cc/kg/day (SD 22.38) vs. 121.71 cc/kg/day (SD 24.12), *p* 0.74 and 135.00 cc/kg/day (SD 32.30) vs. 138.60 cc/kg/day (SD 17.60), *p* 0.34). However, it was observed that patients diagnosed with BPD 2–3 received lower total intake at 15 days of life (143.60 cc/kg/day (SD 27.61) versus 157.32 cc/kg/day (SD 16.88), *p* < 0.01). This difference persisted after adjusting for gestational age in a multivariate model (OR 0.97 (0.96–0.99)) (Table 3).

Regarding nutrition at discharge, 12 patients (30%) in the BPD 2–3 group and 57 (36%) in the noBPD/1 group were discharged with exclusive breastfeeding (EB), with no differences between the two groups (*p* 0.41).

BPD 2–3 patients had lower weight, height, and head circumference (HC) Z scores at birth and discharge compared to noBPD/1 group (Table 3). Regarding growth, patients with BPD 2–3 showed a greater decrease in the difference in the Z score of height at birth and discharge than noBPD/1 patients (−1.64 vs. −0.43, *p* 0.03). There were no statistically significant differences in the difference in the weight and HC Z score between the BPD 2–3 and noBPD/1 groups (−1.11 vs. −0.84, *p* 0.55; −0.30 vs. 0.14, *p* 0.39).

In addition to nutrition-related variables, other variables related to BPD risk were also analyzed. Low weight for gestational age, need for postnatal corticosteroids, hours of IMV, and days of NIV or oxygen therapy were independently associated with the diagnosis of BPD 2–3 regardless of gestational age. No relationship was found between the remaining variables and BPD (Table 4).

## 4. Discussion

BPD is one of the leading causes of morbidity in preterm infants. Understanding modifiable factors, such as the quality of nutrition, could offer strategies to decrease the incidence and severity of BPD, thereby reducing its impact on preterm infant health and associated costs.

MOM has been shown to have a protective effect on BPD, probably related, among others, to its antioxidant effect [5,20,21,22,23,24]. Villamor et al. support the use of standard pasteurized pooled mature donor milk in the absence of MOM, as it has been shown to be protective against BPD compared to preterm formula [23]. Nevertheless, the difference in the incidence of BPD between patients fed MOM and those fed DM is not conclusively established by the evidence. We did not find any difference in the incidence of grade 2–3 BPD between patients who received donated preterm milk and those who received their MOM. As previously described, the donor milk used in this study comes from donor mothers of preterm infants of similar gestational age and days of life. The absence of significant differences in the diagnosis of BPD when comparing patients who received predominantly MOM and those who received predominantly DM is an encouraging finding that supports the use of PDM as an effective alternative when MOM is not available and suggests a benefit compared to the pooled mature milk from term mothers used in standard milk banks.

When analyzing the different macronutrients of donated breast milk, no differences in the composition of milk were observed between the noBPD/1 group and BPD 2–3 group, except for colostrum proteins, and this effect disappeared when adjusting for gestational age and birth weight Z score. Studies by Thiess et al. and Fang et al. indicate that lower protein intake is associated with an increased risk of BPD [25,26]. Similarly, Uberos Fernández et al. [27] describe an increased risk of impaired pulmonary function tests in patients receiving inadequate protein/energy intake in the first week of life. In breast milk, the lower the gestational age, the higher the protein content of the colostrum, as a natural adaptation to the nutritional needs of the most immature newborn. In our institution, donated preterm colostrum with a higher protein content has been assigned to patients with a higher risk of developing BPD when MOM is not available, such as patients with a lower z-score of birth weight. The findings of our study, added to the existing previous literature, suggest that the optimization of nutritional intake in patients with BPD does not depend exclusively on the composition of macronutrients from PDM but also on other micronutrients and fortifiers that were not analyzed in this study [28].

In relation to fluid intake, no differences were observed between patients with BPD 2–3 and noBPD/1 at 3 and 7 days. However, patients diagnosed with BPD 2–3 received fewer fluids at 15 days of age, and this difference was maintained when adjusting for gestational age. The recommendation to implement a water restriction of 130–140 cc/kg/day in preterm infants at risk of BPD [29] makes it difficult to achieve the goals for adequate nutritional intake, which is the main cause of extrauterine growth restriction in these patients [30]. In this context, current guidelines suggest prioritizing appropriate nutritional targets (120–150 kcal/kg/day and 3.5–4 g/kg/day protein) and growth over water restriction [31], recommendations which were applied in this study and which are achieved by adapting the donated milk to their gestational age, thus achieving a higher caloric content than with mature donated milk, as is common practice in standard milk banks.

In the BPD 2–3 group, both exclusive enteral nutrition and the initiation of fortifier supplementation occurred later than in the group with noBPD/I. However, this difference was not maintained when adjusted for gestational age, which may indicate that in patients of lower gestational age, longer times are established to reach enteral exclusively and to initiate fortification with adequate tolerance. These results are consistent with previous studies, which indicate that BPD 2–3 patients and patients of lower gestational age achieved exclusive enteral nutrition later [32,33].

Despite an adequate nutritional intake according to the recommendations of the ESPGHAN [34], it was found that patients with BPD 2–3 had lower growth than the noBPD/1 group, especially in relation to length. Other authors have also observed lower postnatal growth in BPD 2–3 patients [35,36,37]. It has also been observed that a higher caloric intake of energy through enteral feeding is related to an acceleration in the rate of weight gain and a decrease in postnatal growth retardation in preterm infants with bronchopulmonary dysplasia [38].

At hospital discharge, no differences were observed in the incidence of BPD between patients with exclusive breastfeeding (EB), mixed breastfeeding (MB), or artificial breastfeeding (AB). As the indication for donor breast milk (in the absence of the mother’s own breast milk) in patients with severe bronchopulmonary dysplasia is maintained until one or two weeks before discharge, it is not possible to assess the change in diet as this occurred in the final phase of their admission. Therefore, it would be interesting to analyze the impact of the type of nutrition at discharge on respiratory morbidity in the early years of life.

When analyzing the factors associated with BPD in our sample, it has been observed that low weight for gestational age, the need for postnatal corticosteroid administration, hours of invasive mechanical ventilation (IMV), and days of non-invasive mechanical ventilation (NIMV) or oxygen therapy were related to the diagnosis of BPD 2–3, regardless of gestational age. In other studies, risk factors for BPD also include low weight for gestational age and duration of mechanical ventilation, similar to our findings [39,40]. However, in other research, male sex, late sepsis, patent ductus arteriosus, and advanced resuscitation have also been shown to increase the risk of BPD, which have not been observed in our study [41,42,43].

In addition, breastfeeding not only protects against BPD but also reduces the risk of subsequent hospital admissions when administered during the first 6 months of life, with exclusive breastfeeding demonstrating particularly significant benefits [44]. Therefore, it appears essential to provide training during the hospitalization of premature infants to support continued breastfeeding once they are discharged. In addition, multicenter long-term studies are essential to determine the importance of BPD and the protective effect of certain preventive strategies, such as breastfeeding [45].

In summary, this study provides relevant information on the protective role of MOM in the development of BPD, highlighting pasteurized premature donor milk as an alternative to MOM in cases where it is not available. In this context, the approach to BPD must be comprehensive, considering multifactorial aspects and promoting preventive strategies to reduce the severity and incidence of BPD among premature newborns.

## 5. Limitations

The study has some limitations, including its single-center and retrospective design, which may limit the generalizability of the results to wider populations. Data were collected from patients’ electronic medical records and the personalized nutrition unit management program. However, some important data were either missing or incomplete. It should be noted that, due to the retrospective nature of the study, it was not possible to calculate macronutrient intakes for all patients. Therefore, macronutrient comparisons between the groups of noBPD/1 and BPD 2–3 were made using the composition of PDM. At the time this study was conducted, MOM was not analyzed according to the protocol, so there are insufficient data to include MOM macronutrients in the study. Similarly, the analysis of transitional milk could not be included because there was no record of the analysis. Additionally, the study focused only on macronutrients and did not consider the potential impact of other factors, such as micronutrients and bioactive compounds present in donor milk, which could also influence BPD outcomes. Another factor to consider is that, in our unit, most patients received PDM at some point during their admission, especially in the first few days when their own mother’s milk is not yet available. In this context, groups were formed by comparing those who received more than 50% of MOM and more than 50% of PDM, excluding exclusive MOM as another group.

## 6. Conclusions

This study found no differences in the diagnosis of BPD between patients who received predominantly MOM and those who received predominantly PDM. There were also no differences in nutrition during the first days and at discharge between patients with and without BPD, and there was no association between the different macronutrients of the DM and the diagnosis of BPD 2–3. However, further studies are needed to assess the protective effect of diet on the development and severity of BPD.

## Figures and Tables

**Table 1:** Baseline characteristics of the samples.

	NoBPD/1 (*n* = 159)	BPD 2–3 (*n* = 40)	Total (*n* = 199)	*p*
GA *	29.25 (1.90)	26.32 (1.99)	28.67 (2.24)	<0.01
Sex, % male	81 (51%)	22 (55%)	103 (52%)	0.65
Weight	1224.45 (333.32)	781.28 (218.70)	1135.37 (360.25)	<0.01
Weight percentile	46.69 (27.83)	36.20 (28.25)	44.50 (28.26)	0.04
Weight Z Score	−0.17 (1.01)	−0.59 (1.32)	−0.26 (1.09)	0.03
Length	37.66 (3.51)	33.19 (3.29)	36.76 (3.90)	<0.01
Length percentile	40.07 (26.97)	31.41 (27.00)	38.33 (27.13)	0.07
Length Z score	−0.34 (0.97)	−0.72 (1.21)	−0.42 (1.03)	0.04
HC *	26.75 (2.35)	23.56 (1.98)	26.10 (2.61)	<0.01
HC percentile *	47.19 (27.50)	35.63 (26.01)	44.84 (27.54)	0.02
HC Z Score *	−0.09 (1.06)	−0.52 (1.05)	−0.18 (1.07)	0.02
IUGR *	36 (23%)	8 (20%)	44 (22%)	0.72
SGA *	20 (13%)	11 (28%)	31 (16%)	0.02
Antenatal corticosteroids	90 (57%)	17 (43%)	107 (54%)	0.11
Chorioamnionitis	26 (16%)	9 (23%)	35 (18%)	0.36
Cesarean	90 (57%)	19 (48%)	109 (55%)	0.44
Ventilation at delivery	98 (62%)	34 (85%)	132 (66%)	<0.01
Surfactant administration	77 (48%)	29 (73%)	106 (53%)	<0.01
Postnatal corticosteroids	6 (38%)	18 (45%)	24 (12%)	<0.01
IMV *	52 (33%)	35 (88%)	87 (44%)	<0.01
Hours of IMV *	38.50 (101.39)	442.64 (474.74)	120.15 (281.55)	<0.01
NIMV/days of oxygen *	30.43 (23.03)	72.93(27.05)	39.08 (29.35)	<0.01
PDM *	142 (89%)	30 (75%)	172 (86%)	0.05
DM > 50% *	87 (55%)	21 (53%)	108 (54%)	0.95
NEC *	8 (5%)	5 (12%)	13 (6.5%)	0.04
IVH > II *	18 (11%)	10 (25%)	28 (14%)	0.01
PDA *	49 (31%)	23 (58%)	72 (36%)	<0.01
LOS *	29 (18%)	21 (53%)	50 (25%)	<0.01
Oxygen at discharge	0 (0%)	10 (25%)	10 (50%)	<0.01

Categorical variables are expressed as *n* (%) and compared using the chi-square test; quantitative variables are expressed as mean (SD) and compared using the Student’s *t*-test. * GA: gestational age. * HC: head circumference. * IUGR: intrauterine growth restriction. * SGA: small for gestational age. * IMV: invasive mechanical ventilation. * NIMV: non-invasive mechanical ventilation. * PDM: pasteurized preterm donor milk. * NEC: necrotizing enterocolitis. * LOS: late-onset sepsis. * PDA: patent ductus arteriosus.

**Table 2:** Relationship of the different components of PDM and bronchopulmonary dysplasia.

Calostrum	PDM	142 (89%)	30 (75%)	172 (86%)	0.05	
		NoBPD/1 (*n* = 142)	BPD 2–3 (*n* = 30)	Total (*n* = 172)	*p*	aOR (IC)
	Lactose	6.70 (0.34)	6.65 (0.45)	6.69 (0.36)	0.47	0.68 (0.23–1.99)
	Fat	2.83 (0.78)	2.78 (1.01)	2.82 (0.83)	0.72	0.97 (0.57–1.63)
	Protein	2.02 (0.29)	2.24 (0.37)	2.06 (0.32)	<0.01	4.16 (1.12–15.47)
	K Calories	60.37 (7.59)	60.51 (9.61)	60.40 (7.98)	0.92	1.04 (0.95–1.06)
Mature milk	PDM > 50%	87 (55%)	21 (53%)	108 (54%)	0.95	
		NoBPD/1 (*n* = 87)	BPD 2–3 (*n* = 21)	Total (*n* = 108)	*p*	aOR (IC)
	Lactose	7.02 (0.25)	7.18 (0.23)	7.05 (0.25)	0.01	7.27 (0.68–78.15)
	Fat	3.44 (0.59)	3.49 (0.81)	3.45 (0.64)	0.78	1.04 (0.45–2.41)
	Protein	1.62 (0.23)	1.73 (0.37)	1.64 (0.26)	0.09	3.92 (0.49–31.41)
	K Calories	64.81 (8.61)	67.05 (8.22)	65.22 (8.55)	0.31	1.03 (0.95–1.11)

The different macronutrients (lactose, fats, proteins, and calories) are expressed in g/100 mL. Categorical variables are expressed as *n* (%) and compared using the chi-square test. Quantitative variables are expressed as mean (SD) and compared using the Student’s *t*-test; aOR: gestational age-adjusted odds ratio, multivariate model (in parentheses, 95% confidence interval); PDM: pasteurized preterm donor milk.

**Table 3:** Nutritional factors and anthropometry at birth and discharge in noDBP/1 and BPD 2–3 groups.

	NoBPD/1 (*n* = 159)	BPD 2–3 (*n* = 40)	*p*	aOR (IC)
FI 3rd day * (mL/kg)	121.71 (24.12)	123.10 (22.38)	0.74	1.00 (0.99–1.01)
FI 7th day * (mL/kg)	138.60 (17.60)	135.00 (32.30)	0.34	0.99 (0.98–1.01)
FI 15th day * (mL/kg)	157.32 (16.88)	143.60 (27.61)	<0.01	0.97 (0.96–0.99)
FI 30th day * (mL/kg)	10.34 (8.69)	17.28 (14.14)	<0.01	1.01 (0.98–1.05)
Start fortifying (days of life)	10.96 (6.91)	16.62 (8.92)	<0.01	1.03 (0.98–1.10)
At Admission				
Weight (g)	1224.45 (333.32)	781.28 (218.70)	<0.01	0.99 (0.98–0.99)
Weight Z score	−0.17 (1.01)	−0.59 (1.32)	0.03	0.47 (0.32–0.69)
Length (cm)	37.66 (3.51)	33.19 (3.29)	<0.01	0.83 (0.72–0.97)
Length Z score	−0.34 (0.97)	−0.72 (1.21)	0.04	0.53 (0.35–0.81)
HC * (cm)	26.75 (2.35)	23.56 (1.98)	<0.01	0.66 (0.50–0.88)
HC Z score *	−0.09 (1.06)	−0.52 (1.05)	0.02	0.52 (0.33–0.81)
At discharge				
Weight (g)	2468.26 (456.62)	3106.75 (622.45)	<0.01	1.01 (1.01–1.01)
Weight Z score	−1.03 (1.07)	−1.72 (2.59)	0.01	0.80 (0.60–1.08)
Length (cm)	45.40 (5.85)	47.64 (3.36)	0.02	1.12 (0.97–1.29)
Length Z score	−0.75 (1.49)	−2.34 (3.10)	<0.01	0.72 (0.56–0.93)
HC * (cm)	32.98 (2.13)	34.51 (1.75)	<0.01	1.31 (1.09–1.56)
HC Z score *	0.01 (1.16)	−0.86 (2.89)	<0.01	0.88 (0.69–1.13)

Quantitative variables are expressed as mean (SD) and compared using the Student’s *t*-test; aOR: gestational age-adjusted odds ratio, multivariate model (in parentheses, 95% confidence interval); * FI: fluid volume intake, ml/k; * HC: head circumference.

**Table 4:** Relationship of perinatal variables and BPD adjusted by gestational age.

	aOR (IC)	*p*
Sex, % male	0.60 (0.25–1.42)	0.24
Weight Z score	0.47 (0.33–0.70)	<0.01
Length Z score	0.53 (0.35–0.81)	<0.01
HC Z score *	0.52 (0.33–0.81)	<0.01
IUGR *	1.48 (0.54–4.03)	0.44
SGA *	7.25 (2.32–22.69)	<0.01
Antenatal corticosteroids	0.93 (0.52- 1.66)	0.81
Corioamnionitis	1.08 (0.39–3.01)	0.87
Cesarean	0.69 (0.29–1.64)	0.40
Ventilation at delivery	1.77 (0.62–5.05)	0.28
Surfactant application	1.35 (0.55–3.31)	0.52
Postnatal corticosteroids	4.55 (1.34–15.53)	0.01
IMV *	4.98 (1.64–15.15)	<0.01
IMV Hours *	1.005 (1.002–1.007)	<0.01
NIMV/Days of oxygen *	1.06 (1.03–1.08)	<0.01
NEC *	2.38 (0.57–9.95)	0.23
IVH III/IV *	0.71 (0.22–2.31)	0.57
PDA *	1.08 (0.43–2.73)	0.87
LOS *	0.72 (0.28–1.87)	0.50

Logistic regression comparison; aOR: gestational age-adjusted odds ratio, multivariate model (in parentheses, 95% confidence interval). * HC: head circumference. * IUGR: intrauterine growth restriction. * SGA: small for gestational age. * NEC: necrotizing enterocolitis (>Grade 2 Bell). * IVH III/IV: grade III or IV intraventricular hemorrhage. * LOS: late-onset sepsis. * PDA: patent ductus arteriosus. * IMV: invasive mechanical ventilation. * NIMV: non-invasive mechanical ventilation.

## Data Availability

All study data are provided in the manuscript. Detailed methods and additional data are available upon request from the corresponding authors.
